# Evaluation of the Relationship Between Serum and Urine Ferritin Level of Low Birth Weight Infants

**DOI:** 10.30699/IJP.2022.546540.2807

**Published:** 2022-08-13

**Authors:** Hassan Bazmamoun, Soheila Narimani, Maryam Shokouhi, Hossein Esfahani, Ali Reza Soltanian, Ali Reza Rastgoo Haghi

**Affiliations:** 1 Department of Pediatric Gastroenterology, Hamadan University of Medical Sciences, Hamadan, Iran; 2 Department of Pediatrics, Hamadan University of Medical Sciences, Hamadan, Iran; 3 Department of Neonatal-Perinatal Medicine, Hamadan University of Medical Science, Hamadan, Iran; 4 Department of Pediatric Hematology/Oncology, Hamadan University of Medical Science, Hamadan, Iran; 5 Department of Biostatistics, Hamadan University of Medical Sciences, Hamadan, Iran; 6 Department of Pathology, Hamadan University of Medical Science, Hamadan, Iran

**Keywords:** Low birth weight infants, Serum ferritin, Urine ferritin

## Abstract

**Background & Objective:**

Iron deficiency before birth or in infancy can cause long-term behavioral and neurological disorders. Measuring serum ferritin is an effective way to diagnose iron deficiency but requires significant blood volume from a low birth weight infant. Therefore, the present study was performed to investigate the relationship between serum and urinary ferritin levels in low birth weight infants.

**Methods:**

In this cross-sectional study, 76 infants weighing less than 2500 g were studied. To measure serum ferritin level, 1.5 mL of blood and to measure urinary ferritin level, at least 1 mL of urine was collected from each infant. Then the results were compared. Data analysis was performed using SPSS software version 16, and the significance level was considered less than 0.05.

**Results:**

Out of 76 neonates studied, 51.3% were boys, and 80.3% were premature infants. The mean birth weight of infants was 2056.31±318.74 g, and the mean serum and urinary ferritin levels were 134.77±72.35 and 85.55±70.97 ng, respectively. There was a statistically significant relationship between serum and urinary ferritin levels. Also, serum ferritin and urinary ferritin levels had a statistically significant relationship with birth weight and gestational age. The higher the birth weight as well as the age at birth, the higher the serum ferritin and urinary ferritin.

**Conclusion:**

According to the findings of this study, measurement of urinary ferritin level can be used as a noninvasive tool for iron deficiency screening in low birth weight infants instead of serum ferritin level.

## Introduction

Iron is one of the most important micronutrients that play an important role in functioning of all vital systems of the body, especially the brain. When iron deficiency occurs in the prenatal or neonatal period, it can cause long-term behavioral and neurological disorders. (1, 2). Iron deficiency at birth is more common among high-risk groups, such as infants with low birth weight compared to gestational age, premature infants with very low birth weight, infants of diabetic mothers, and infants of mothers who smoke (3-6). Clinical signs of iron deficiency anemia include paleness, irritability, premature fatigue, muscle weakness, increased heart and respiration rate, enlarged heart and spleen, and decreased alertness, and can slow the child's normal growth and development. Therefore, timely diagnosis and treatment of iron deficiency are very important, especially in infants and children (7-10).

Current iron deficiency screening methods focus more on the diagnosis of anemia. But anemia is a relatively late effect of iron deficiency and probably does not occur until the brain's iron deficiency causes a neurological defect in the infant. (7). 

Ferritin is made from iron and a protein called Apo ferritin in the cell cytoplasm and is the best indicator for measuring iron stores in the body. According to studies, measuring serum ferritin is an effective method of identifying iron deficiency (11-14). However, the use of ferritin in low birth weight infants is limited due to the significant volume of blood required for phlebotomy, indicating the need for alternative screening in infants at higher risk for iron deficiency. Elevated serum ferritin is seen in cases such as pregnant women with preterm labor and preterm PROM, as well as an acute phase reactant (15, 16)

Given the above, this study was performed to investigate whether measuring urinary ferritin levels can be used as a noninvasive tool for screening low-weight infants for iron deficiency.

## Material and Methods

This cross-sectional study was performed in 2021 on 76 low birth weight infants (less than 2500 g) under 28 days who were born in Fatemieh Hospital in Hamadan. Neonates were randomly selected by the easy sampling method.

Inclusion criteria included age less than 28 days, weight less than 2500 grams at birth, and absence of infectious, inflammatory, liver, and kidney diseases in the infants. 

Exclusion criteria included parental dissatisfaction and the impossibility of sampling the neonate for any reason.

After obtaining informed parental consent, 1.5 mL of blood from each infant was collected and centrifuged to measure serum ferritin. The supernatant was then transferred to the tube for testing within two hours. Serum ferritin level was measured by chemiluminescent immunoassay method (Mindray kit, China) and the results were reported in ng/mL. Within 24 hours after sampling, at least 1 mL of urine was collected using a urine bag to measure urinary ferritin. Urine ferritin was measured by a method similar to serum ferritin (CL-Mindray kit, China), and the results were reported in ng/mL.

Demographic data of patients were collected using a questionnaire.

Using statistical indices of mean and standard deviation, quantitative variables such as age, serum, and urine ferritin level were described. Contingency tables and percentages were used to describe qualitative data. Pearson's correlation coefficient was used to determine the relationship between serum and urine ferritin levels. Data analysis was performed using SPSS version 16 (SPSS Inc., Chicago, IL., USA), and the significance level was considered less than 0.05.

This study was financially supported by the vice chancellor for research and technology of Hamadan University of Medical Sciences (no: 9903201692). It was approved by the Ethics Committee of Hamadan University of Medical Sciences with code IR.UMSHA.REC.1399.246**.**

## Results

Of the 76 neonates, 39 (51.3%) were male, and 61 (80.3%) were preterm. The mean birth weight of infants was 2056.31±318.74 g, and the mean serum and urine ferritin levels were 134.77±72.35 and 86.55±70.97 ng, respectively.

There was a statistically significant relationship between serum and urine ferritin levels. There was also a statistically significant relationship between serum and urine ferritin levels with birth weight and gestational age. There was a statistically significant relationship between birth weight and gestational age (Table 1).

Serum and urine ferritin levels were higher in male infants than in female infants, but this difference was not statistically significant (Table 2). 

Serum and urine ferritin levels in term neonates were higher than in preterm neonates, which was statistically significant. (Table3).

**Table 1:** Comparison of serum and urine ferritin levels with gestational age in low birth weight infants

Gestational age	Birth weight	Urine ferritin	Serum ferritin	Variable	
**0.363** ^**^	0.119	0.902^**^	*1*	Pearson's correlation coefficient	Serum ferritin
**0.001**	0.3	0.000		P-value	
**0.331** ^**^	0.052	1		Pearson's correlation coefficient	Urine ferritin
**0.004**	0.65			P-value	
**0.353** ^**^	1			Pearson's correlation coefficient	Birth weight
**0.002**				P-value	
**1**				Pearson's correlation coefficient	Gestational age
				P-value	

**Table 2:** Serum and urine ferritin levels in low birth weight infants

P-value	Mean± STD	Number	Variable	
**0.4**	141.58 ± 70.18	39	Boy	Serum ferritin
	127.58 ± 74.84	37	Girl	
**0.18**	97.21 ± 75.37	39	Boy	Urinary ferritin
	75.32 ± 45.15	37	Girl	

**Table 3:** Serum and urine ferritin levels in term and preterm low birth weight infants

P-value	Mean± STD	Number	Variable	
**0.000**	148.88 ± 71.1	15	Term	Serum ferritin
	77.39 ± 44.48	61	Preterm	
**0.000**	98.25 ± 71.86	15	Term	Urine ferritin
	38.99±42.78	61	Preterm	

## Discussion

The results of this study showed that there is a statistically significant relationship between serum and urine ferritin levels. There was also a statistically significant relationship between serum and urine ferritin levels with birth weight and gestational age. 

Unfortunately, in the literature review, few similar studies were found in this area for comparison.

In a pilot study by Baher* et al.* (2019), serum and urine ferritin levels in a limited number of healthy adult men (n=5), healthy term infants (n=5), and premature infants (n=8) as well as children with high ferritin levels due to iron overload or liver disorders (n=6), was measured. The results showed that serum and urinary ferritin levels were significantly correlated, similar to our study's results. The researchers suggested that measuring urinary ferritin levels could be used as an alternative screening method to diagnose iron deficiency in cases where blood sampling is limited (17).

In a study by Gerday E *et al.* (2021), performed at the PICU on 49 infants at risk for iron deficiency, there was a statistically significant relationship between urine and serum ferritin levels which was in line with the results of our study (18). One of the shortcomings of this study, as noted by the article's authors, is that the study focused on a selected subset of NICU patients at risk for iron deficiency, while in our study, there was no such limitation.

In another study by Ishikawa* et al.* (1982), conducted on healthy adults in Japan, there was a statistically significant relationship between serum and urine ferritin levels. Also, serum ferritin in healthy men (4.1 ng/mL) was significantly higher than in healthy women (1.8 ng/mL). This difference was also present in their urine ferritin levels (19). In our study, serum and urine ferritin levels were higher in male infants than in female infants, but this difference was not statistically significant.

In the study of Lipschitz* et al.* (1980), urine ferritin was measured by the immune-radiometric method in healthy volunteers and patients with various hematological diseases. The average concentration of urine ferritin in normal individuals was 2.2 micrograms per liter, which was about 3% of serum ferritin level. An increase in urine ferritin level of up to 45 μg/L was observed in patients with malignancy, which was accompanied by a proportional increase in serum ferritin so that the urine ferritin level was still, on average 7% of serum ferritin. The highest levels of urine ferritin (mean 170 μg/L) were seen in chronic hemolytic anemia. In these patients, urine ferritin was disproportionately higher than serum ferritin (mean≈82%). Therefore, the result was that, in normal people and in malignant patients, the origin of urine ferritin is different because there is a link between urine ferritin and reticuloendothelial iron storage. In this case, the most likely source of urine ferritin is iron in renal tubular cells, which is in balance with the body's iron stores (20). 

## Conclusion

urine ferritin measurement can be used as a noninvasive tool to screen iron deficiency in low birth weight infants instead of serum ferritin.

## Conflict of Interest

The authors declared no conflict of interests.

## Funding

This study was financially supported by the vice chancellor for research and technology of Hamadan University of Medical Sciences, Iran, with grant No. 9903201692.
